# Cerebrospinal fluid lipidomic biomarker signatures of demyelination for multiple sclerosis and Guillain–Barré syndrome

**DOI:** 10.1038/s41598-020-75502-x

**Published:** 2020-10-27

**Authors:** Mária Péter, Wanda Török, Anna Petrovics-Balog, László Vígh, László Vécsei, Gábor Balogh

**Affiliations:** 1 Institute of Biochemistry, Biological Research Centre, Szeged, 6726 Hungary; 2 Department of Neurology, University of Szeged, Szeged, 6725 Hungary; 3 MTA-SZTE Neuroscience Research Group, University of Szeged, Szeged, 6725 Hungary

**Keywords:** Lipidomics, Neurological disorders, Multiple sclerosis, Mass spectrometry

## Abstract

Multiple sclerosis (MS) and Guillain**–**Barré syndrome (GBS) are demyelinating disorders affecting the central nervous system and peripheral nervous system (PNS), respectively. Cerebrospinal fluid (CSF) is one of the most valuable sources of diagnostic biomarkers in neurological diseases. In the present study high sensitivity shotgun mass spectrometry was used to characterise the CSF lipidome of patients with MS, GBS and controls with non-demyelinating diseases. The quantification of 222 CSF lipid molecular species revealed characteristic changes in the absolute and relative lipid concentrations in MS and GBS compared to the controls. For the GBS group, the fourfold elevation in the total lipid content was a discriminatory and a newly identified feature of PNS demyelination. In contrast, in MS, the accumulation of the myelin-derived cerebrosides represented a specific feature of demyelination. As a common feature of demyelination, we identified upregulated levels of lipid metabolic intermediates. We found strong positive correlation between total protein content and lipid concentrations in both diseases. By exploring the CSF lipidome we demonstrate usefulness of broad-range shotgun lipidomic analysis as a fast and reliable method of biomarker discovery in patients with demyelinating neurological disorders that might be a valuable diagnostic complement to existing examinations.

## Introduction

Multiple sclerosis (MS) is a chronic inflammatory disease, where demyelination specifically affects the central nervous system (CNS)^1^. MS is one of the most common diseases of the CNS, the incidence rate is 30 cases per 100,000 people globally but in Europe it reaches 80 cases per 100,000. The symptoms show substantial degree of heterogeneity with autonomic, visual, motor and sensory problems being the most common^1,2^. Although its cause is unknown, it is assumed to occur as a result of complex genetic, epigenetic and environmental factors. The diagnosis is based on the integration of clinical, imaging and laboratory findings. The majority of patients experience a relapsing–remitting type of MS, however, over time half of these patients enter a progressive phase. Currently, there is no known cure for MS, but research is ongoing looking for more effective and tolerable treatments.

Guillain**–**Barré syndrome (GBS) is a postinfectious, acute immune-mediated polyradiculoneuropathy, where demyelination occurs in the peripheral nervous system (PNS)^3,4^. The incidence of GBS is rare, the rate is 1–2 cases per 100,000 people. GBS is thought to be an autoimmune disease, and the onset of symptoms is preceded in two-thirds of cases by a bacterial or viral gastroenteritis or respiratory infection^5^, Campylobacter jejuni, Epstein–Barr virus, cytomegalovirus and Zika virus being among the most common infective agents. Curiously, recent reports suggested the possible association between acute COVID-19 infection and GBS^6^, therefore the incidence of GBS during outbreak of COVID-19 infection can be increased. GBS is characterised by acute onset and a rapidly progressing ascending type of motor weakness, paraesthesia which reaches maximum severity within 4 weeks, where ca. 25–30% of patients require artificial ventilation^7^. The diagnosis is based on clinical history, nerve conduction studies and analysis of the cerebrospinal fluid (CSF). To alleviate its course, intravenous immunoglobulin therapy or plasmapheresis is used. With appropriate supportive care the prognosis of GBS is good and leads to full recovery in the majority.

A common pathological feature in MS and GBS is the leakage of the blood–brain/blood-CSF or blood-nerve barriers^8–11^. CSF is one of the most valuable and accessible sources of diagnostic biomarkers in neurological diseases. CSF is not simply an ultrafiltrate of plasma but fills and flushes the transcellular space of brain and spinal cord. Therefore, both the abnormal plasma infiltration and the appearance of myelin degradation products, as major consequences of demyelination, can be tracked by the analysis of CSF. It is known that the plasma and brain lipidomes display characteristic differences. For example, high levels of the storage lipids cholesteryl ester and triglyceride are plasma characteristics^12,13^, whereas sulfatides, gangliosides and the myelin-specific cerebrosides, also referred to as hexosylceramides, are typical brain lipids^14,15^. At lipid species level, the brain is rich in disaturated, monounsaturated and docosahexaenoic acid (22:6)-containing phospholipid species such as phosphatidylcholine PC(16:0/16:0), PC(16:0/18:1) and phosphatidylethanolamine PE(18:0/22:6), respectively^15,16^. In contrast, plasma lipid profile is known for the abundance of linoleic acid (18:2)-containing species such as PC(16:0/18:2) or arachidonic acid (20:4)-containing species like PC(16:0/20:4)^12,13^. Given these characteristic differences, CSF lipidomics might provide valuable information with regard to the origin as well as the underlying mechanisms of demyelination.

From bioanalytical point of view, CSF is a challenging biological fluid due to its low 15 µg/mL total lipid content^17^, which is approximately 300-fold lower than that of plasma^12,17^. The development of high sensitivity mass spectrometry-based approaches^18^ enabled the examination of the CSF lipidome^19^. However, insofar reports focused mainly on dementia^20–22^, and comprehensive CSF lipidomic reports dealing with MS and GBS are few and incomplete.

Substantial symptom heterogeneity in MS and fast disease progression in GBS necessitate exploration of new rapid methods of biomarker discovery. In this study we applied a direct injection-based mass spectrometry method for the fast and comprehensive assessment of the CSF lipidome that allows for the correlation of lipid data with available clinical parameters and identification of differences in the CSF lipidomic patterns of demyelination in MS and GBS compared to non-demyelinating diseases.

## Results

### Study population

Of the 118 neurological patients recruited from the Hungarian population in the city of Szeged, 77 proved eligible to participate in an exploratory retrospective CSF analysis. Table 1 provides a summary of demographic data and clinical parameters. 24 patients with MS represented demyelination affecting the CNS. At the time of sampling, all patients were at the beginning of their disease and 92% were diagnosed with relapsing–remitting MS with a fairly narrow disability range (1–4) quantified by the Expanded Disability Status Scale (EDSS). Only one of the two patients with primary progressive modality of MS displayed a high EDSS value of 8.5. Nineteen patients with GBS represented demyelination affecting the PNS, and 34 patients with non-demyelinating disorders were considered as controls. In accordance with global data, we observed 67% female predominance in the MS group and a higher proportion of males in the GBS group (58%). In the GBS group, CSF total protein content exceeded the normal upper limit of 0.45 mg/mL in 84% of cases, which is in agreement with the consensus that an increased CSF protein content with normal cell count is considered a hallmark of GBS^3^. We detected strongly elevated albumin index in the GBS group with 73% of cases exceeding the normal maximum value of 9. In patients with MS, intrathecal IgG synthesis leads to the elevation of IgG to albumin ratio in CSF compared to serum^23^. The IgG index exceeded the normal limit of 0.7 in 63% of cases, and the IgG blot was positive in 96% of cases.

**Table 1:** Summary of demographic and clinical data. Data represent median (interquartile range). ^#^Upper cut-off value of normal range; *NA* not available (50% of data missing or not determined); ^†^15/19 data available. *Significance was determined by Van der Waerden post-hoc tests (for age, CSF protein and cell nr) or by Chi square test (for sex) and accepted for *P* < 0.05 (control versus MS or GBS). *Control* non-demyelinating diseases; *MS* multiple sclerosis; *GBS* Guillain–Barré syndrome; *CSF* cerebrospinal fluid; *EDSS* expanded disability status scale; *HFGS nadir/discharge* Hughes Functional Grading Scale at the lowest point of disease/at leaving for home or rehabilitation.

	Control	MS	GBS
Patient nr	34	24	19
Sex (female%)	65	67	42
Age	60 (55–68)	39 (36–46)*	54 (45–63)*
CSF protein (mg/mL, cut-off 0.45^#^)	0.37 (0.31–0.44)	0.40 (0.36–0.47)	0.97 (0.62–1.33)*
CSF cell nr	1.2 (0.7–2.0)	6.8 (1.5–18.1)*	1.0 (0.7–2.5)
Albumin index (cut-off 9^#^)	NA	5.7 (4.3–7.1)	25.0 (9.4–31.3)^†^
IgG index (cut-off 0.7^#^)	NA	0.83 (0.62–1.10)	0.56 (0.51–0.62)^†^
IgG blot (pos%)	NA	96	NA
EDSS sampling	NA	2.5 (1.5–3.0)	NA
HFGS nadir	NA	NA	4.0 (3.0–4.0)
HFGS discharge	NA	NA	3.0 (2.0–3.0)

### CSF shotgun lipidomics

To identify lipid biomarker signatures of demyelination, we applied high sensitivity, high resolution shotgun mass spectrometry-based lipidomic method (Supplementary Fig. S1). Using 250 μL of CSF, the lipidomic workflow consisted of a two-phase liquid–liquid lipid extraction in the presence of an extraction standard, reconstitution of the extract in the infusion solvent containing a set of mass spectrometry internal standards, addition of additives for positive and negative ion mode measurements and direct injection-based electrospray ionisation mass spectrometry assessment of extracted lipids on an Orbitrap Elite instrument equipped with an automated chip-based delivery system. Survey acquisitions were performed at high mass resolution. Lipid species were identified by LipidXplorer software^24^. To present lipidomic data we calculated both absolute quantities (lipid nmol/CSF mL) and relative levels (mol% of polar lipids) (Supplementary Tables S1–S2). Relative quantification was made by comparing the integrated signal intensities of identified peaks with those of the corresponding internal standards, whereas absolute quantification was made based on the amount of the extraction standard. To resolve fatty acyl composition in glycerophospholipids, tandem MS2 or MS3 fragmentation experiments were performed. Our lipidomic platform allowed the broad-range coverage of the CSF lipidome within minutes; we identified and quantified 222 lipid molecular species in 19 lipid classes including glycero- and glycerophospholipids, sphingolipids and cholesteryl ester (for basic lipid structures and an illustration of CSF lipid class composition see Supplementary Fig. S2).

### Characterisation of the CSF lipidome with regard to its origin

**Figure 1:**
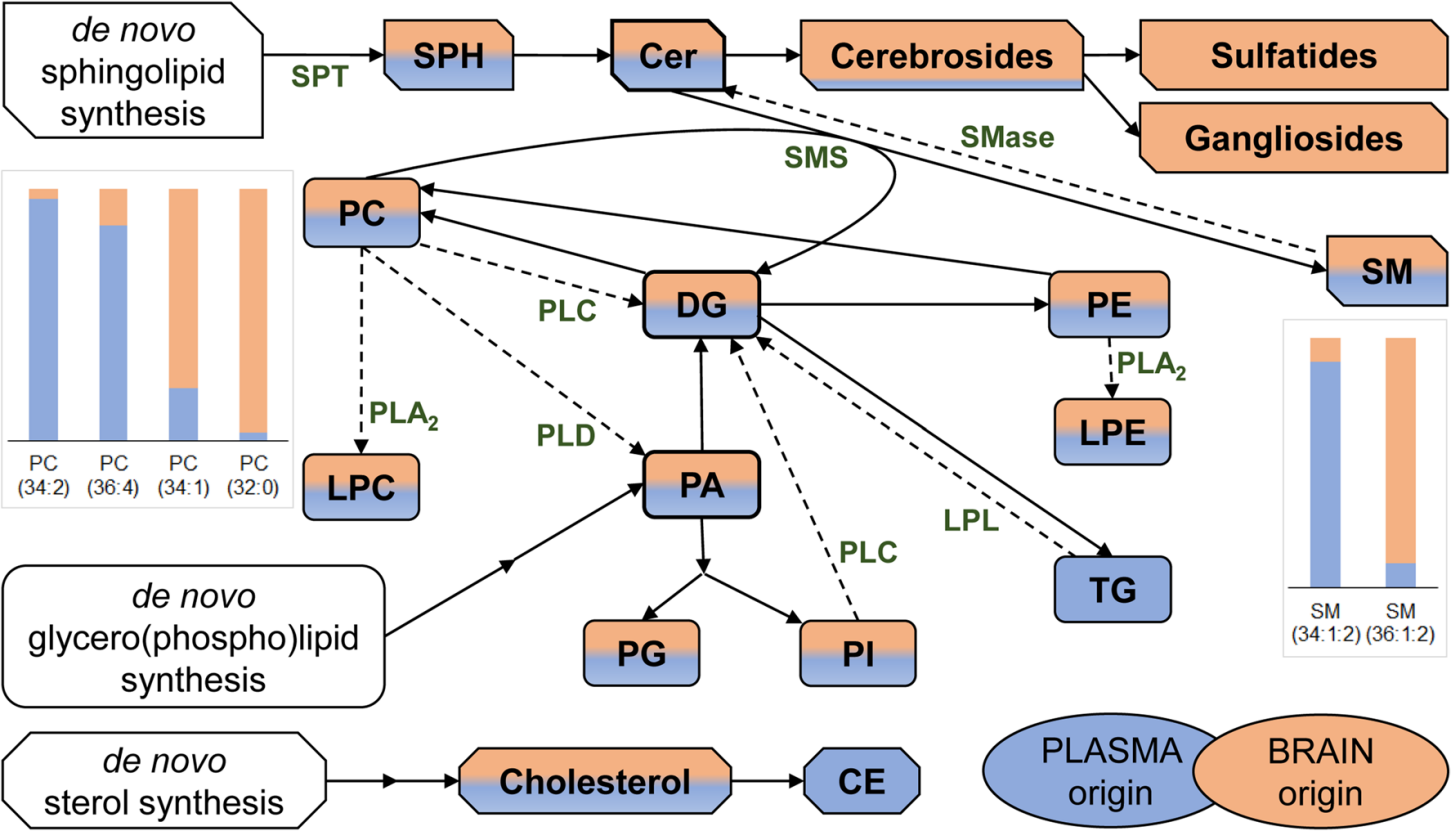
Metabolism and the origin of CSF lipids. Blue and orange fill indicate plasma and brain/myelin origin, respectively. For lipid classes, colouring illustrates but does not quantify the origin contribution. Solid arrows display anabolic steps, whereas dashed arrows indicate those catabolic steps that are discussed in the present study. *CE* cholesteryl ester; *Cer* ceramide; *DG* diglyceride; *LPC* lysophosphatidylcholine; *LPE* lysophosphatidylethanolamine; *PA* phosphatidic acid; *PC* phosphatidylcholine; *PE* phosphatidylethanolamine; *PG* phosphatidylglycerol; *PI* phosphatidylinositol; *SM* sphingomyelin; *SPH* sphingoid base; *TG* triglyceride. *LPL* lipoprotein lipase; *PLA*_*2*_*/PLC/PLD* phospholipase A_2_/C/D; *SMase* sphingomyelinase; *SMS* SM synthase; *SPT* serine palmitoyltransferase. We note that SPH, higher gangliosides (GM1, GD, GT) and cholesterol were not assessed in the present study.

Combining our data with the major characteristics of the plasma and brain lipidomes, in Fig. 1 we provide a brief overview of CSF lipid metabolism with indication of the origin of different lipids. The abundance of the storage lipids cholesteryl ester and triglyceride in the CSF reflects plasma-specific features, whereas the sizeable amounts of sulfatides and cerebrosides reflect brain-specific components (Supplementary Table S1 and Supplementary Fig. S2). Phosphatidylcholine (PC) and sphingomyelin (SM) represent the most abundant glycerophospho- and sphingolipid classes in CSF, respectively. PC and SM are also major lipid classes in both plasma and brain, nevertheless, their species compositions are characteristically different. At lipid species level, the relatively high level of PC(32:0, 16:0/16:0) and PC(34:1, 16:0/18:1) indicate brain-derived analytes in CSF, whereas the abundance of PC(34:2, 16:0/18:2) and PC(36:4, 16:0/20:4) resemble the lipid profile found in plasma (Fig. 1 and Supplementary Table S1; for fatty acyl-resolved structures see Supplementary Table S3). Similarly, palmitic acid-containing SM(34:1:2, d18:1/16:0) originates mainly from plasma, whereas the stearic acid-containing SM(36:1:2, d18:1/18:0) derives largely from the brain (Fig. 1).

### Disease-specific lipidomic biomarkers

**Figure 2:**
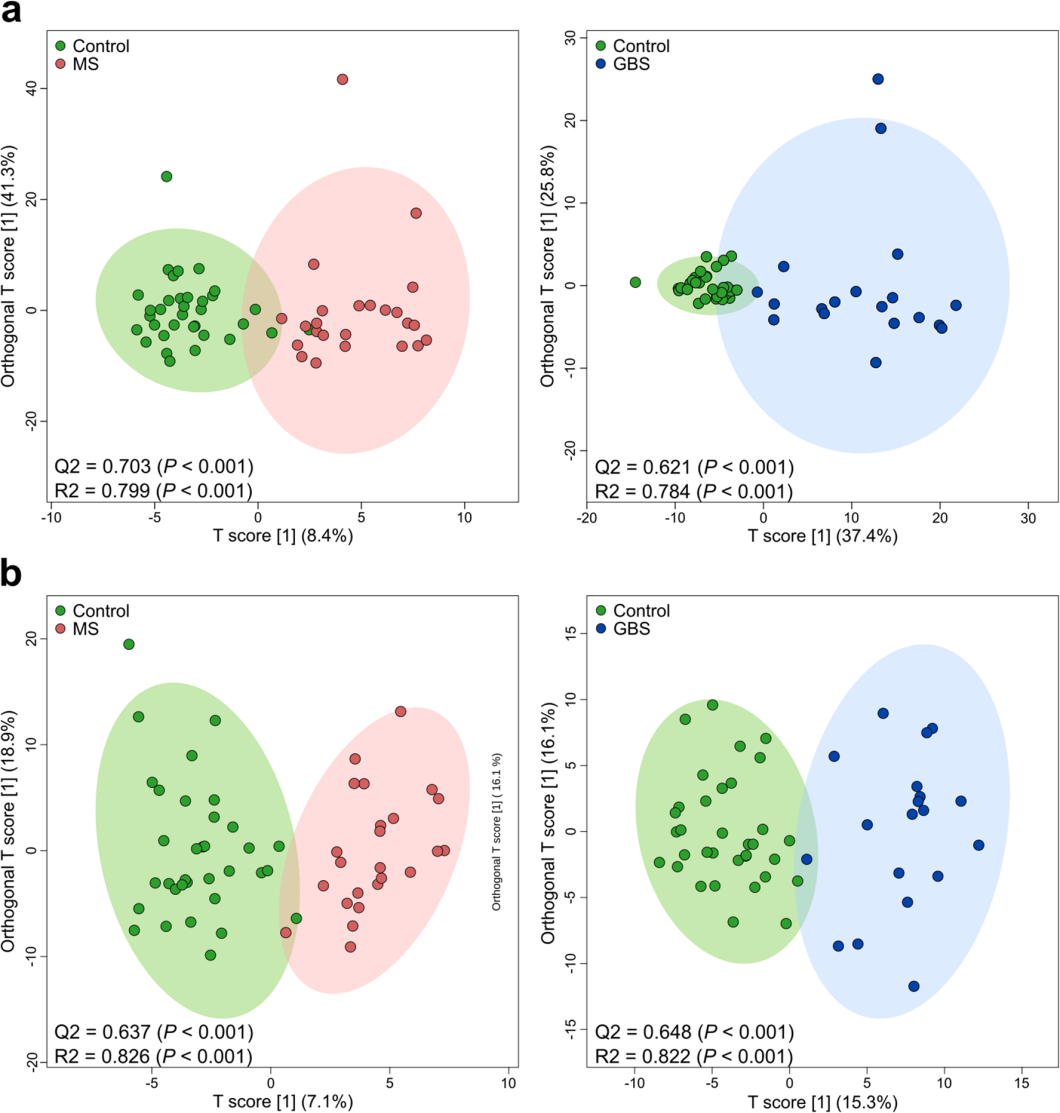
OPLS-DA analysis of lipidomic datasets. OPLS-DA scores plots are shown based on (**a**) absolute and (**b**) relative lipid levels; coloured circles display 95% confidence regions; control, n = 34; MS, n = 24; GBS, n = 19. Q2 and R2 values represent the predictability and goodness of fit, respectively, with indication of *P* values of random permutation tests (n = 1000) in parenthesis. *Control* non-demyelinating diseases; *MS* multiple sclerosis; *GBS* Guillain–Barré syndrome; *OPLS-DA* orthogonal partial least squares discriminant analysis.

Next, we performed multivariate statistical analysis on our lipidomic data. Absolute and relative lipid concentration values were subjected to OPLS-DA; the group separations, shown in the scores plots for control versus MS and control versus GBS groups, were validated by permutation tests that returned good predictability (Q2) and high goodness of fit (R2) with *P* < 0.001 values in all cases (Fig. 2a,b). The well distinguishable clusters indicated that characteristic changes occurred both in the lipid content (Fig. 2a) and in the lipid composition (Fig. 2b) of CSF in both diseases.

**Figure 3:**
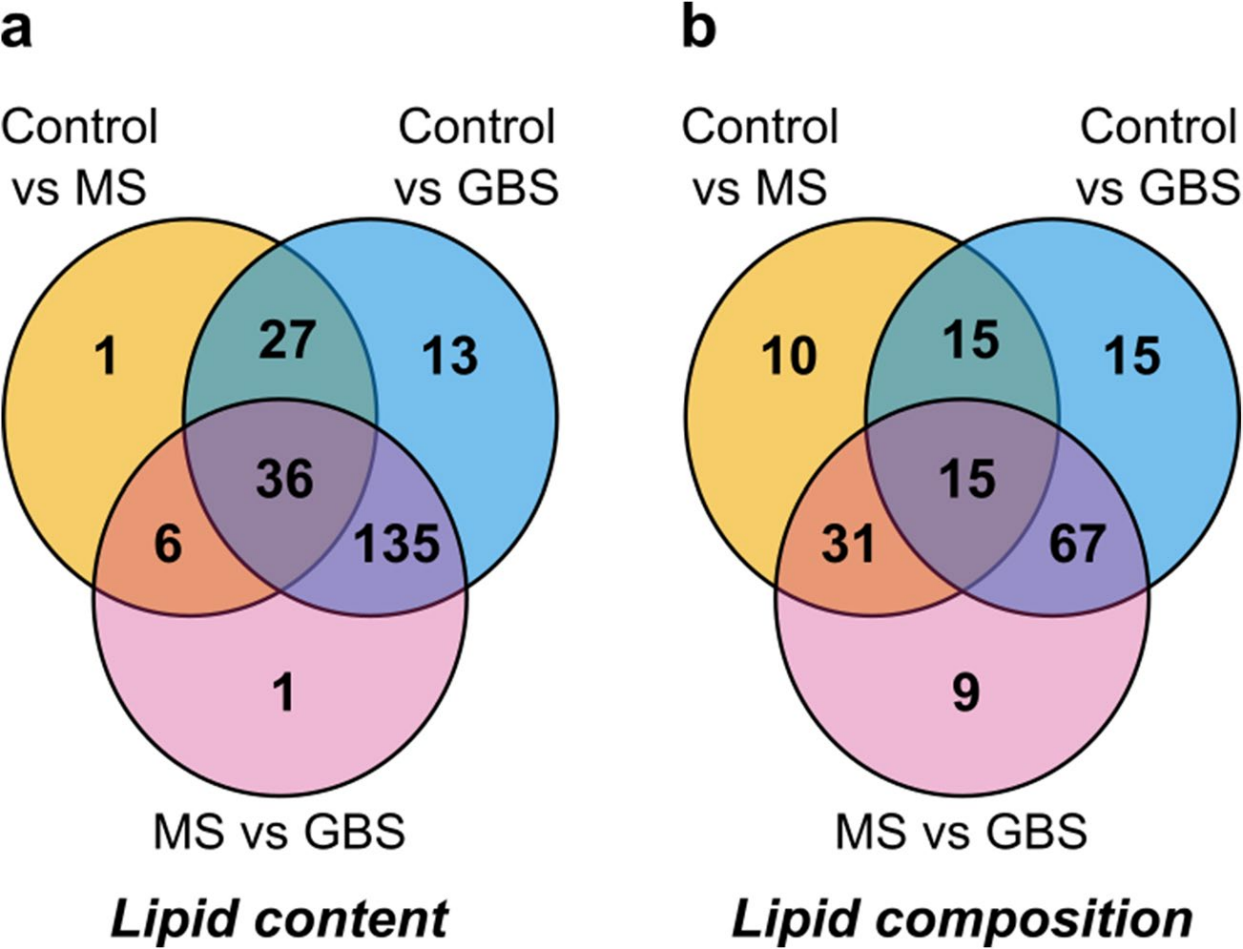
Comparison of the CSF lipid molecular species patterns for the control, MS and GBS groups. Venn diagrams display the number of statistically different components for (**a**) lipid content expressed as nmol/mL CSF and for (**b**) lipid composition expressed as mol% of polar lipids; control, n = 34; MS, n = 24; GBS, n = 19. Significance was determined based on Van der Waerden post-hoc tests and accepted at *P* < 0.05 and *q* < 0.05. *Control* non-demyelinating diseases; *MS* multiple sclerosis; *GBS* Guillain–Barré syndrome.

**Figure 4:**
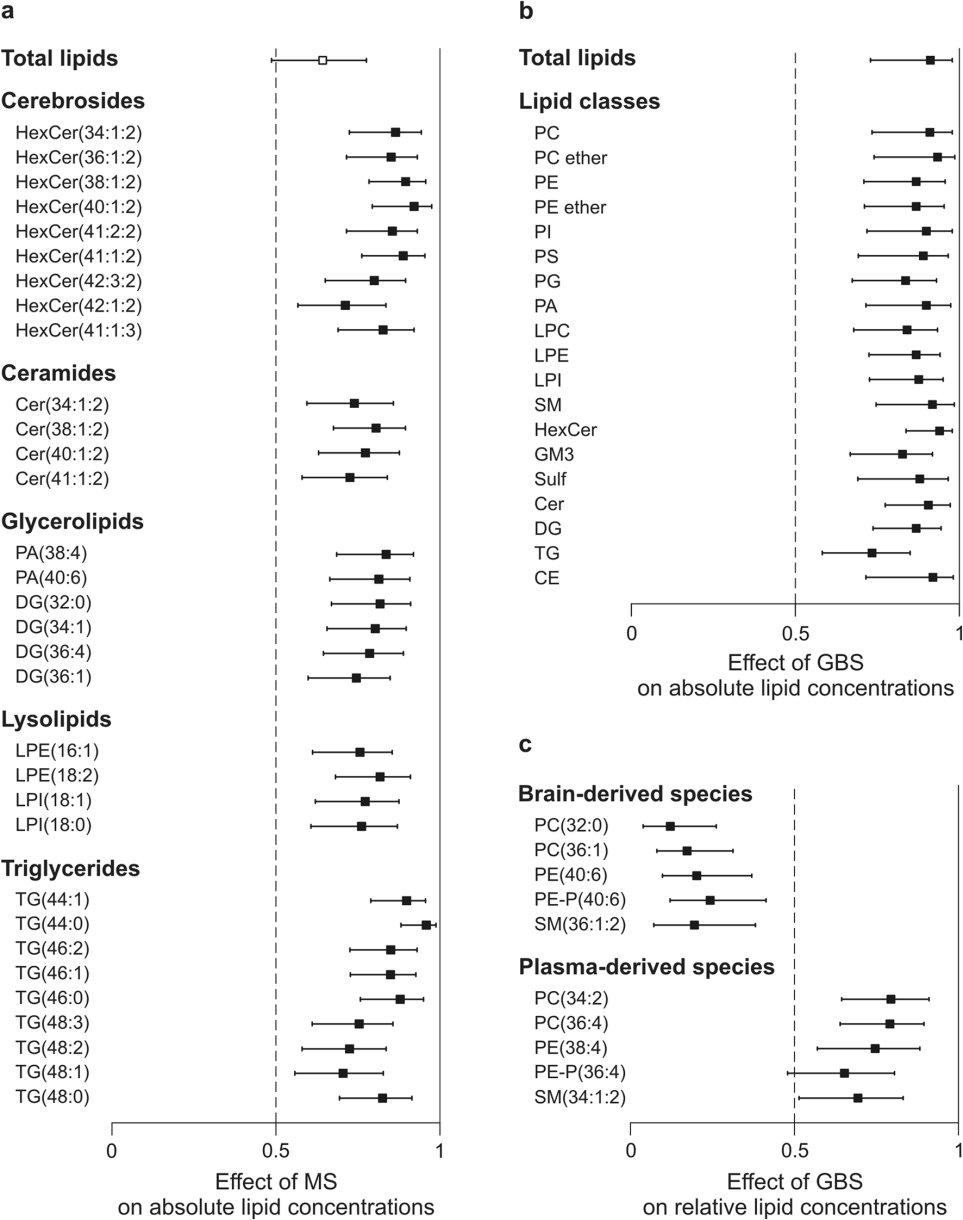
Effect of MS or GBS on CSF lipid levels. Forest plots represent the effect sizes A and 95% confidence intervals for absolute concentration values (nmol/CSF mL) (**a**) between the control and MS groups, (**b**) between the control and GBS groups and (**c**) for relative concentration values (mol% of polar lipids) between the control and GBS groups; control, n = 34; MS, n = 24; GBS, n = 19. A equals the area under a receiver operating characteristic curve (AUC); A = 0.5 represents no change (dashed lines), whereas 0 and 1 represent maximal effects, decrease and increase, respectively. Filled squares indicate significance determined based on Van der Waerden post-hoc tests and accepted for *P* < 0.05 and *q* < 0.05. *Control* non-demyelinating diseases; *MS* multiple sclerosis; *GBS* Guillain–Barré syndrome. *PC* diacyl phosphatidylcholine; *PC ether* alkyl/alkenyl-acyl phosphatidylcholine, *PE* diacyl phosphatidylethanolamine; *PE ether* alkyl/alkenyl (PE-P)-acyl phosphatidylethanolamine; *PI* phosphatidylinositol; *PS* phosphatidylserine; *PG* phosphatidylglycerol; *PA* phosphatidic acid; *LPC* lysophosphatidylcholine; *LPE* lysophosphatidylethanolamine; *LPI* lysophosphatidylinositol; *SM* sphingomyelin; *HexCer* cerebroside; *GM3* GM3 ganglioside; *Sulf* sulfatide; *Cer* ceramide; *DG* diglyceride; *TG* triglyceride; *CE* cholesteryl ester. Forest plots were created by R 3.6.3 (https://www.R-project.org).

Subsequently, we compared the molecular species patterns for the control, MS and GBS groups using Van der Waerden post-hoc tests for pairwise multiple comparisons (Supplementary Tables S1–S2). Venn diagrams shown in Fig. 3, visualized the number of statistically different lipid species. Considering absolute lipid concentrations (nmol/CSF mL, Fig. 3a), the result showed that 36 lipid components distinguished all study groups, whereas 70 and 211 lipid species altered significantly in the CSF of patients with MS and GBS compared to the controls, respectively. For compositional data (mol% of polar lipids, Fig. 3b), altogether 162 analytes changed significantly in any pairwise comparison; 71 and 112 of these separated MS and GBS from the control group, respectively. In order to identify relevant alterations, we grouped the changes according to lipid classes, lipid metabolic pathways, and brain- or plasma-specific features. We calculated the nonparametric effect size A and illustrated selected results on forest plots (Fig. 4).

Sphingolipid metabolism is known to have a characteristic role in the CNS^25^. Because myelin is rich in sphingolipids, especially cerebrosides, sphingolipids may play a marker role in demyelination. Although the total lipid content did not change, our lipidomic data revealed an elevation in almost all cerebroside and in several ceramide (Cer) species in the MS group compared with the controls (Fig. 4a and Supplementary Table S2). The glycerolipid metabolism showed alterations too; levels of phosphatidic acid, diglyceride, lysolipid and low molecular weight triglyceride species were increased (Fig. 4a and Supplementary Table S2).

The total lipid content of CSF from patients with GBS showed a statistically significant fourfold increase compared to the controls (from 14.7 (11.9–17.2) to 57.5 (33.3–79.7) nmol/CSF mL, *P* = 2E−09). The elevation included practically all measured lipid classes (Fig. 4b) and lipid species (Supplementary Table S2) but its extent varied considerably. Cholesteryl ester, PC and SM displayed the largest differences, and the mostly elevated species were dominantly plasma-derived lipid components (Supplementary Table S4). The increase in lipid content, expressed as fold change (ratio of GBS to control), also revealed large variation between lipid classes (from 1.8 to 11.4, Supplementary Table S5). The highest fold increases were detected for lysophosphatidylethanolamine (11.4), lysoPC (10.1) and Cer (6.6). In addition, well distinguishable changes occurred in the composition of CSF in the GBS group compared with the controls. These changes were characterized by the elevation in the relative levels of several plasma-derived components accompanied by the reductions in brain-derived species (Fig. 4c).

### Correlation of clinical and lipidomic data

**Figure 5:**
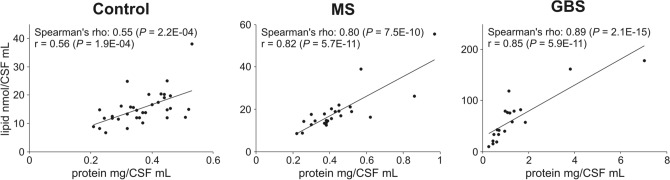
Correlation between CSF total protein and total lipid contents. Circles in scatter plots represent individual samples; control, n = 34; MS, n = 24; GBS, n = 19. Solid lines represent linear regression. Spearman’s rho values display partial correlation coefficients with control for age. r values represent Pearson correlation coefficients. *Control* non-demyelinating diseases; *MS* multiple sclerosis; *GBS* Guillain–Barré syndrome.

Next, we analysed the potential correlations between clinical and lipidomic features using the Spearman’s rank correlation analysis. In the absence of age-matched controls, we calculated partial correlation coefficients to control for the effect of age. Nevertheless, we note that age has negligible effect on lipid metabolite levels (see e.g. the scatter plot for total lipid versus age in Supplementary Fig. S3). CSF total protein content displayed very strong positive correlation (r ≥ 0.8) with the total lipid content in the MS and GBS groups, whereas it was moderate (0.5 < r < 0.7) in the control group (Fig. 5). In addition, we observed strong correlations (r > 0.7) between total protein content and absolute lipid concentrations at both the lipid class and lipid species level. The number of cases with strong correlation was 50 and 149 in the MS and GBS group, respectively, whereas in the control group it never exceeded the correlation value of 0.7 (Supplementary Table S6). Lipid concentration strongly correlated with albumin index as well but there was no association with IgG index.

We also assessed whether lipid levels were associated with disease-specific disability scores. For the MS group, we did not detect statistically significant correlation with EDSS. This might be due to the homogeneity of the MS group dominated by patients with relapsing–remitting MS (92% of cases) with a fairly narrow EDSS range (1–4). However, we note that the EDSS value of 8.5, found in a patient with primary progressive MS, appeared to associate with markedly higher levels for several lipid species throughout the whole lipidome. For the GBS group, several relative lipid concentration values, including sphingo-, glycero and glycerophospholipid species, displayed moderate correlation with Hughes Functional Grading Scale (HFGS) disability scores measured at nadir and/or discharge. Noteworthy, correlations were positive for plasma-derived phosphatidylethanolamine, lysolipid, diglyceride and triglyceride components, whereas negative correlation was found for brain-specific sulfatide and GM3 ganglioside species (Supplementary Table S7).

**Figure 6:**
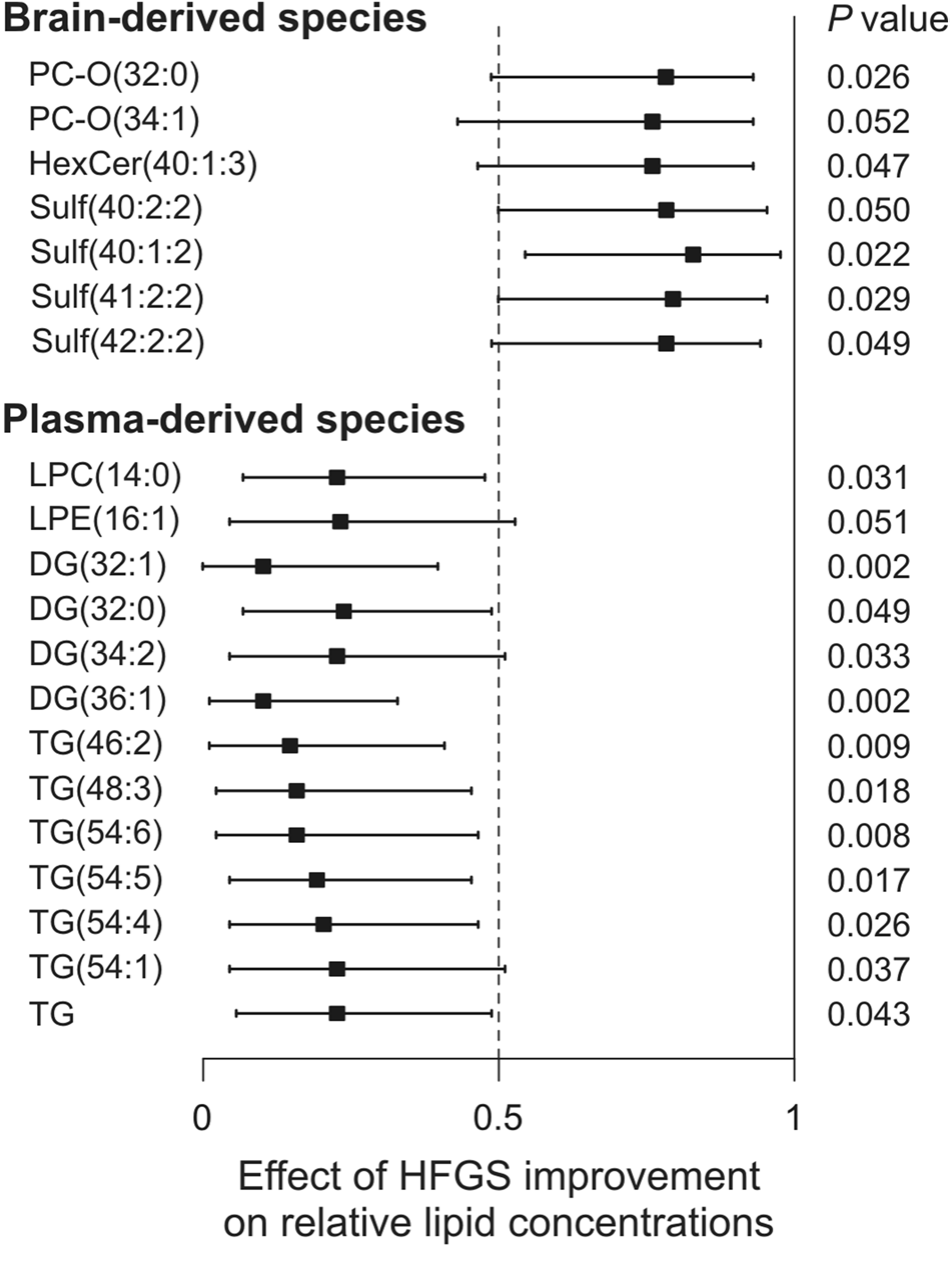
Association of relative lipid levels with HFGS improvement. Forest plots represent the effect sizes A and 95% confidence intervals for relative lipid concentration values (mol% of polar lipids) between non-improving (ΔHFGS = 0, n = 8) and improving (ΔHFGS = 1–3, n = 11) GBS subgroups. A equals the area under a receiver operating characteristic curve (AUC); A = 0.5 represents no change (dashed lines), whereas 0 and 1 represent maximal effects, decrease and increase, respectively. *P* values were determined by Van der Waerden test. *GBS* Guillain–Barré syndrome; *HFGS nadir/discharge* Hughes Functional Grading Scale at the lowest point of disease/at leaving for home or rehabilitation; *PC-O* alkyl-acyl phosphatidylcholine; *HexCer* cerebroside; *Sulf* sulfatide; *LPC* lysophosphatidylcholine; *LPE* lysophosphatidylethanolamine; *DG* diglyceride; *TG* triglyceride. Forest plots were created by R 3.6.3 (https://www.R-project.org).

Finally, we calculated the differences between HFGS determined at nadir and discharge for each patient. Based on these results, we divided the patients into two groups, a non-improving (ΔHFGS = 0, n = 8) and an improving group (ΔHFGS 1–3, n = 11). We detected no statistically significant differences between these categories with respect to the clinically diagnostic biomarker parameters (protein content, albumin index, IgG index). Interestingly, brain-derived sulfatide and PC species showed higher relative levels, whereas plasma-derived lysolipid, diglyceride and triglyceride species displayed lower values in patients in the clinically improving group (Fig. 6). It suggests that the lower the relative accumulation of plasma-derived lipids in the CSF of a patient with GBS, the better the chance of clinical recovery.

## Discussion

In the present study we report about a rapid and comprehensive lipid species analysis of the CSF lipidome, an insofar understudied area in CSF biomarker discovery. We presented the applicability of our method in this exploratory study that was aimed to better understand the role of lipidomic biomarkers in the pathology of demyelination in the CNS and PNS. There are only a few reports in the literature that analysed CSF lipids in MS or GBS. These studies were targeted either on SM analysis in demyelinating disorders of the PNS^26^, on the assessment of the sphingolipidome^27^ or the global lipidome with a fatty acid-targeted approach^28^ in patients with MS, or on the polar metabolome from patients with MS^29^ and GBS^30^.

It is a generally accepted view that the blood–brain/blood-CSF barrier is compromised in both MS^31^ and GBS^32^. The consequences, i.e. the enhanced plasma infiltration and the augmented appearance of myelin-derived lipids in CSF are well distinguishable features by lipidomic analysis given the characteristic differences in the plasma and brain lipidomes. In general, we detected a distinctive change in the absolute and relative lipid amounts in MS and GBS as compared to controls with non-demyelinating disorders. This elevation suggests a characteristic alteration in lipid metabolism and/or lipid transport due to CNS or peripheral site of demyelination. The strong association between the total protein content and lipid levels represents a new piece of information for both diseases. For the GBS group, the massive elevation in lipid amounts was a distinctive and newly recognized feature of PNS demyelination. In addition to the highly elevated albumin index, the robust accumulation of plasma-derived lipids (cholesteryl ester, plasma-derived species of PC and SM) as well as the imbalance in the composition of plasma-derived versus brain-derived lipid species confirmed the blood–brain/blood-CSF barrier damage and the abnormal leakage of plasma into the CSF. The elevation in SM agrees well with recently published results where targeted CSF lipid analysis revealed significantly higher SM content in patients with GBS compared to those with non-demyelinating disorders^26^. In the MS group, we could not observe increase in albumin index. This could be in agreement with reports that propose albumin index may not be an accurate measure of blood–brain barrier dysfunction in MS^31^. Nevertheless, at the level of the lipidome, the enrichment of plasma-specific triglyceride species might be an indication of enhanced plasma leakage into the CSF of patients with MS.

On the other hand, the accumulation of sulfatides, ganglioside GM3 and cerebrosides in the GBS group, as well as the enrichment of cerebrosides in the MS group are indicative of demyelination. Considering the overall alterations in the CSF lipidomes, the selective accumulation of the myelin-specific cerebrosides seems to be a characteristic feature of CNS demyelination compared with that in the PNS. An increase in the levels of cerebrosides in the CSF of patients with MS has already been reported^27^. In agreement with those findings, the palmitic acid-containing cerebroside species HexCer(34:1:2) was one of the best predictive markers for MS as compared to controls in our study too.

As a common feature of demyelination, we detected upregulated levels of lipid metabolic intermediates in both disease groups. The distinctively high-fold increases of lysoPC, lysophosphatidylethanolamine and Cer in patients with GBS, as well as the increase of Cer, phosphatidic acid and diglyceride in patients with MS might point to a general activation of lipid degradation. Based on these data, at the site of the blood–brain/blood-CSF/blood-nerve barrier damage, activation of different lipases could occur as a result of the inflammatory response. The elevation of Cer species in the MS group agrees well with previous findings in the literature. The Cer species were elevated in the CSF of patients with MS, and it was causatively linked to inducing neuronal mitochondrial dysfunction and axonal damage^33^. Other studies have demonstrated that Cer can be important mediator of oligodendrocyte death in the MS brain^34^. Furthermore, the activity of acid sphingomyelinase, which generates Cer from SM (Fig. 1b), increased in astrocytes in active MS lesions^35^. It was also shown that acid sphingomyelinase plays a significant role in myelin repair and its inhibition promotes remyelination^36^. On the other hand, a novel observation was reported that the elevation of Cer and sphingosine occurred in the experimental model of MS via de novo Cer generation due to activation of serine palmitoyltransferase^37^. The increase in diglyceride in both MS and GBS can be the result of either phospholipase C activation, which removes the headgroup of glycerophospholipids, or lipoprotein lipase activation, which hydrolyses triglyceride (Fig. 1b). It was shown that the elevation and activity of lipoprotein lipase activity may be part of an acute response to scavenge and reutilize myelin-derived lipids in degenerating peripheral nerves^38^ as well as by microglia^39,40^. Similarly, the elevation in phosphatidic acid might be due to phospholipase D induction, which cleaves the choline moiety from the headgroup of PC (Fig. 1b). PLD1 ablation markedly reduced symptomatology in experimental allergic encephalomyelitis raising the possibility that PLD1 inhibition may provide a useful approach in MS therapeutics^41^. In addition, phosphatidic acid-induced demyelination led to the development of pronounced peripheral neuropathy in a mouse model^42^. In line with these findings, we observed elevated levels of several lysolipid species in both GBS and MS compared to the controls. This might be the result of phospholipase A_2_ activation, which removes a fatty acyl tail from glycerophospholipids (Fig. 1b). In addition, lysoPC is a known potent demyelinating agent either when injected into the spinal cord and other CNS regions or when applied to the PNS^43–45^. Since remyelination in the PNS is efficient and quick but limited by the presence of the demyelinating agent^46^, treatments to lower lysoPC might be considered in GBS therapeutics.

Altogether, several events that occur during and after demyelination might simultaneously contribute to the complex changes in the lipidomic profile in the CSF of patients with MS or GBS. These may include alterations in intrathecal lipid metabolism as it was demonstrated by the drastic change in the Cer species in patients with active MS lesions^35^. Concomitant changes in plasma and CSF lipid composition present an important subject for further investigation. Indeed, the disturbance in the plasma lipidomic profile was observed in patients with GBS compared to healthy controls^47^. Moreover, the contribution of changes in the transport selectivity of lipids from plasma into the CSF or altered re-uptake selectivity of lipids from the CSF into blood should also be considered.

Although we could not show correlation between CSF lipid levels and EDSS in patients with MS in the present study, in a previous report, where follow-up data were available, the cerebroside species HexCer(34:1:2) appeared to be a promising candidate biomarker of disease progression in MS^27^. In patients with GBS, the significant correlation between HFGS and relative CSF lipid levels represents new results. In addition, the potential prognostic value of sulfatides or triglyceride can be valuable in a fast progressing disease.

Limitations of the present study include the relatively small size of the study population and that recruitment of patients was from a geographically restricted area of Europe. Nevertheless, the study groups represented well the neurological disorders in focus with regard to basic demographic and clinical parameters. Therefore, our sample was justifiably suitable to perform an exploratory lipidomic study. An additional limitation is the absence of age-matched controls; nevertheless, the correlation analysis showed that age-dependence of lipid metabolite levels was negligible in our cohort. The predictive and especially the prognostic power of the CSF lipidomic analytical results should be verified in a larger cohort study in the future. Assessment of higher gangliosides, parallel plasma lipidomics and correlation of lipidomic data with clinical imaging results could further improve the understanding of the molecular mechanisms behind these demyelinating disorders.

## Conclusion

The dry mass of both CNS and PNS myelin is characterized by a high proportion of lipids (70 to 85%)^14^, therefore, lipid analysis might be of valuable diagnostic value in demyelinating disorders. Here we provided evidence for the massive increase in CSF lipid content as well as for the relative accumulation of plasma-derived over brain-derived lipids as newly identified CSF lipidomic features in patients with GBS. These results revealed that abnormal plasma infiltration into CSF dominates the pathology of PNS demyelination. In contrast, in the MS group, the selective elevation in the amount of myelin-derived cerebrosides reports about a dominantly myelin degradation-driven pathology in the CNS. In addition, our data suggest the activation of different phospholipases, likely as a result of the inflammatory response at the site of the blood–brain/blood-CSF barrier damage. CSF total protein content showed strong positive associations not only with the total lipid content but also with the levels of several individual lipid species in both MS and GBS. This finding provides a good basis for further investigations in which CSF lipidomics together with the analysis of individual CSF proteins might reveal the role of specific lipid-protein interactions in the pathology of demyelination.

The low lipid content, accompanied by relatively high salt concentration, makes CSF a challenging biofluid from lipidomic point of view. Most studies apply chromatography-coupled methods for analytical purposes. Nevertheless, our data demonstrate usefulness of the direct injection-based shotgun strategy that might enable cross contamination-free, high throughput lipidomic analysis with broad-range coverage of the CSF lipidome. CSF shotgun lipidomics can therefore serve biomarker discovery in other neurological diseases beyond MS and GBS.

## Methods

### Standard protocol approvals, registrations and patient consents

The Regional Human Biomedical Research Ethics Committee has approved the analysis conducted in this study (135/2008). All examinations and experiments including lumbar puncture and CSF collection were carried out in accordance with the guidelines of the Declaration of Helsinki and were performed after obtaining written informed consent.

### Participants

Utilizing the CSF depository of the Department of Neurology (University of Szeged, Hungary), we performed retrospective analysis using 118 neurological patients recruited between 2007 and 2017 from the Hungarian population. CSF samples of patients with alcoholic diseases (e.g. Wernicke encephalopathy), acute infections (e.g. viral meningitis, pneumonia, mastoiditis, urosepsis, tuberculosis, Lyme disease), polyneuropathies (e.g. diabetic and alcoholic polyneuropathies, and patients with B12-deficient funicular myelosis), systemic autoimmune diseases (systemic lupus erythematosus, anaemia perniciosa-Hashimoto thyreoiditis), and cancer were excluded from the study. In addition, we diagnosed 9 patients with other demyelinating diseases (clinically isolated syndrome, optic neuromyelitis). However, due to the low case number they could not be handled as a specific diseased control group and were also omitted from the study.

Filtering left 77 participants eligible for this study including 22 patients with relapsing–remitting and 2 patients with primary progressive modality of MS, and 19 patients with the diagnosis of GBS (68.4% AIDP, 26.3% AMAN and 5.3% Miller-Fisher subtypes). Disease modifying treatment was always started after the time of sampling in patients with MS or GBS. 34 patients with non-demyelinating disorders were considered as controls including patients with cerebral infarction (9), syncope (1), transient global amnesia (1), vertigo (2), headache (2), spinal injury (8), CADASIL (1), cerebellar ataxia (1), vascular myelopathy (2), cognitive impairment (3), glaucoma (1), amyotrophic lateral sclerosis (1), pseudodementia (1), and cerebral cavernoma (1). Patients with MS were in the acute relapsing stage of the disease and the diagnosis was confirmed using the McDonald’s diagnostic criteria^48^. The Expanded Disability Status Scale (EDSS; ranging from 0 to 10, higher numbers indicate more severe disability) was used to quantify disability at the time of sampling. Patients with GBS were classified according to the Brighton criteria^49^. The samples were collected within 1–49 days of symptom onset, before major therapeutic interventions (immunoglobulin therapy, plasmapheresis). Motor functional deficits were retrospectively scored at nadir (lowest point of disease) and discharge (leaving for home or rehabilitation) by the Hughes Functional Grading Scale (HFGS; ranging from 0 to 6, higher numbers indicate more severe disability).

### Sample collection and processing

CSF and serum samples were obtained, processed, and analysed according to the international standardized biobanking consensus protocol of the BIOMS-Eu network^50^. Albumin index and IgG index were calculated as albumin_CSF_/albumin_serum_ and (IgG/albumin)_CSF_/(IgG/albumin)_serum_, respectively.

### Lipid extraction

The solvents used for lipid extraction and for mass spectrometry analyses were of liquid chromatographic grade (Merck, Darmstadt, Germany) and Optima LCMS grade (Thermo Fisher Scientific, Bremen, Germany). Lipid standards were obtained from Avanti Polar Lipids (Alabaster, AL, USA) and Nu-Chek-Prep (Elysian, MN, USA). All other chemicals were purchased from Sigma-Aldrich (Steinheim, Germany) and were of the best available grade. Lipids from fresh-frozen CSF samples were extracted by using a modified version of the standard two-phase Folch extraction^51^. The modifications included the formation of a one-phase solvent system (as in the Bligh and Dyer method^52^) before phase separation which ensures extensive lipid extraction. Moreover, we applied pure water instead of 0.2 M KCl upon phase separation induction. Because CSF contains enough salt to keep more polar lipids in the lower organic phase, this modification helped to reduce ion suppression, which is critical in shotgun lipidomics. To 250 μL of native CSF sample, 625 μL methanol containing 0.001% butylated hydroxytoluene (as antioxidant) and 0.5 μg di20:0-PC (as extraction standard) and 313 μL chloroform were added to form one phase (chloroform:methanol:water = 1:2:0.8, by vol.). The mixture was left at room temperature for an hour with periodical vortexing, then 938 μL chloroform and 220 μL water were added for phase separation to occur (final composition of chloroform:methanol:water = 2:1:0.8, by vol.)). After vortexing, the sample was centrifuged at 600 g for 10 min and the lower chloroform phase was evaporated. The residue was reconstituted in 300 μL chloroform:methanol:isopropanol (1:2:1, by vol.) containing a mass spectrometry internal standard mix. The standard mix composition was adjusted to CSF lipid profile (Supplementary Table S8) and was validated by comparison with that of the SPLASH LIPIDOMIX (Avanti Polar Lipids, 330707).

### Mass spectrometry-based shotgun lipidomics

Lipidomic analyses were performed on an LTQ-Orbitrap Elite instrument (Thermo Fisher Scientific). For sample delivery we applied a robotic nanoflow ion source TriVersa NanoMate (Advion BioSciences, Ithaca, NY, USA) using chips with spraying nozzles having a diameter of 5.5 μm. The ion source was controlled by Chipsoft 8.3.1 software. The ionization voltages were + 1.3 kV and − 1.9 kV in positive and negative mode, respectively, and the backpressure was set at 1 psi in both modes. The temperature of the ion transfer capillary was 330 °C. These conditions, together with the applied infusion solvent composition and additive types, ensured degradation-free survey scans as validated previously^53^. For mass spectrometry measurements, to 60 μL reconstituted lipid extract 4 μL dimethylformamide (additive for the negative ion mode, final concentration 6%) or to 30 μL reconstituted lipid extract 34 μL 17.5 mM ammonium chloride (additive for the positive ion mode, final concentration 9 mM) was added. 10 μL of these solutions was injected and data were acquired for 3 min. Acquisitions were performed at the mass resolution R_m/z 400_ = 240,000. All CSF lipid extracts were measured in one batch (within 3 days), and the instrument was fully calibrated before batch processing. A pooled extract was prepared from the control samples and was used as a QC sample injected after every tenth sample^54^; all 8 QC injections fell within the 95% confidence interval after PCA analysis run on test (individual controls) and injection QC samples (Supplementary Fig. S4). Phosphatidylcholine (PC), lysophosphatidylcholine (LPC), sphingomyelin (SM), diacylglycerol (DG), triacylglycerol (TG) and cholesteryl ester (CE) were detected and quantified using the positive ion mode, whereas phosphatidylethanolamine (PE), phosphatidylinositol (PI), phosphatidylserine (PS), the lyso derivatives LPE and LPI, phosphatidic acid (PA), phosphatidylglycerol (PG), ceramide (Cer), hexosylceramide/cerebroside (HexCer), sulfatide (Sulf) and ganglioside GM3 were detected and quantified using the negative ion mode. Lipid species were identified by LipidXplorer software^24^. Identification was made by matching the m/z values of their monoisotopic peaks to the corresponding elemental composition constraints. The mass tolerance was set to 2 ppm. Quantification was made by comparing integrated signal intensities with those of the internal standards after built-in C13 isotopic corrections. To resolve fatty acyl composition in glycerophospholipids, data-dependent tandem MS2 or MS3 fragmentation experiments were performed for PC, PE, PI and PS based on mass lists from survey scans (Supplementary Table S3). Data files generated by LipidXplorer queries were further processed by in-house Excel macros.

To annotate lipid classes and species, we applied the classification systems for lipids^55^. Sum formulas for glycero(phospho)lipids are defined as the lipid class abbreviation followed by the total number of carbons and total number of double bonds for all chains, e.g. PC(34:1), whereas the lipid class(*sn*-1/*sn*-2) format specifies the structures and regiochemistry of fatty acyl side chains, e.g. PC(16:0/18:1). Sum formulas for sphingolipids are defined as the lipid class abbreviation followed by the total number of carbons, total number of double bonds and total number of hydroxyl groups in the long chain base and the fatty acyl moiety, e.g. SM(34:1:2). Sphingosine, SPH(d18:1), was considered as the major sphingoid base in which first the number of hydroxyl groups is designated by a letter (e.g. “d” for two hydroxyls) followed by the number of carbons and double bonds. In higher sphingolipids, the lipid class(sphingoid base/fatty acyl) format specifies the resolved structure, e.g. SM(36:1:2) corresponds to SM(d18:1/18:0).

Lipidomic data are expressed either as absolute quantities (lipid nmol/CSF mL) or as relative levels. Relative levels were calculated as mol% of polar lipids, where polar lipids include glycerophospholipids and sphingolipids but not diglycerides and storage lipids. Absolute concentrations were calculated based on the amount of the extraction standard.

### Statistical analysis

Demographic, clinical, and lipidomic data are presented as median and interquartile range. Because lipidomic data did not fulfil the assumptions of normality (Shapiro–Wilk test) and homoscedasticity (F test), we applied the Van der Waerden test for between-subjects analysis of variance^56^. Subsequently, Van der Waerden post-hoc tests were performed for pairwise multiple comparisons. We calculated the nonparametric effect size index A, which equals the area under a receiver operating characteristic curve (AUC) using the trapezoidal method and estimates the probability that a member of one population scores higher than a member of another population. A = 0.5 represents no change, whereas 0 and 1 represent maximal effects, decrease and increase, respectively^57^. A_1,2_ = 1 − A_2,1_ where the indexes of A refer to the two populations to be compared. A is not sensitive to group sizes and is much more robust to unequal variances or outliers^58^. False discovery rate (*q* value) was determined according to the Storey Tibshirani method^59^. We calculated partial correlation coefficients to describe associations between clinical and lipid data with the control for age using Spearman’s rank correlation. These analyses were performed using R 3.6.3 (R Foundation for Statistical Computing, Vienna, Austria)^60^. Orthogonal partial least squares discriminant analysis (OPLS-DA) on lipidomic datasets was performed with MetaboAnalyst^61^.

## Supplementary information

Supplementary Tables.

Supplementary Figures.
