# Supplementary material for: Cerebrospinal fluid lipidomic biomarker signatures of demyelination for multiple sclerosis and Guillain–Barré syndrome
Source: Sci Rep. 2020 Oct 27;10:18380. doi: 10.1038/s41598-020-75502-x (PMC7592055; doi:10.1038/s41598-020-75502-x)
Supplement: Supplementary file 2 — Supplementary Figures. [file 41598_2020_75502_MOESM2_ESM.pdf]

Supplementary Information

Cerebrospinal fluid lipidomic biomarker signatures of demyelination for multiple sclerosis and Guillain–Barré syndrome

Mária Péter, Wanda Török, Anna Petrovics-Balog, László Vígh, László Vécsei, Gábor Balogh

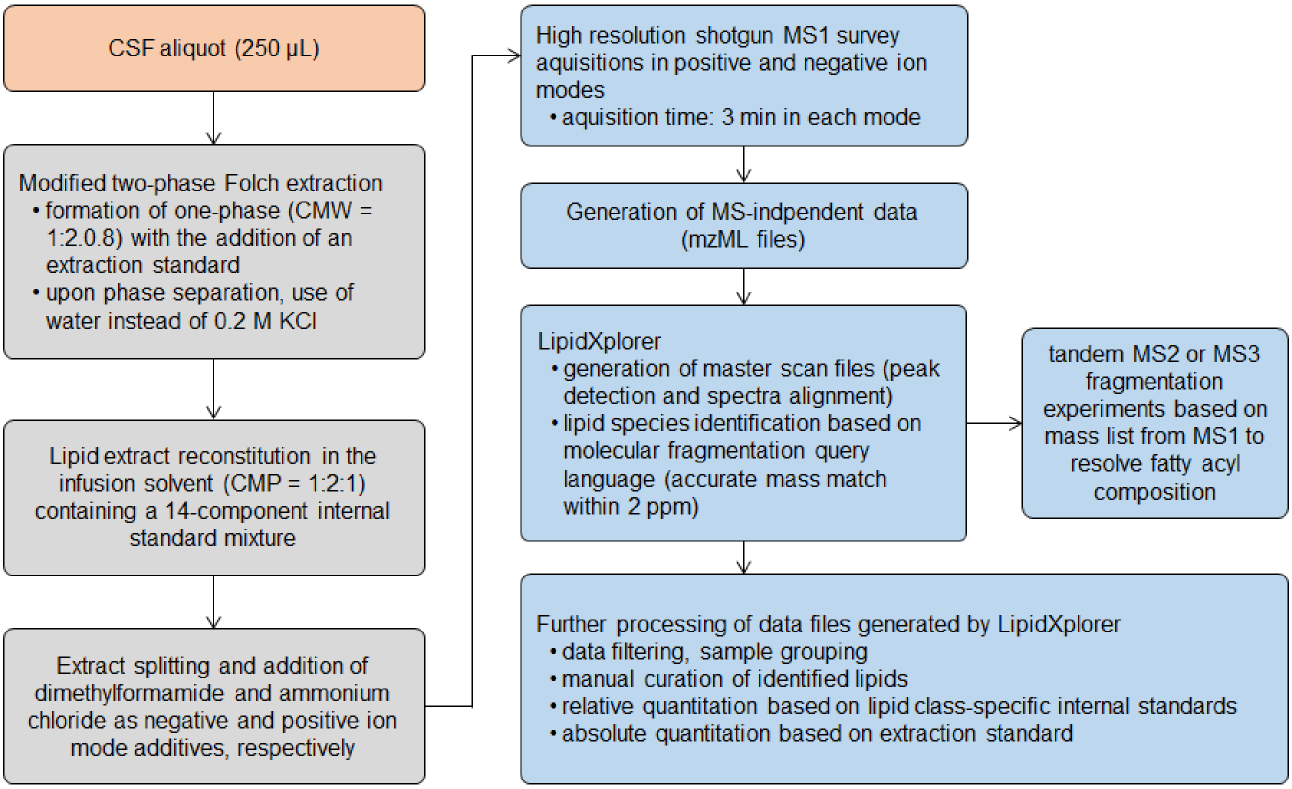

Supplementary Fig. S1. Schematic illustration of the lipidomic workflow. CMW, chloroform-methanol-water; CMP, chloroform-methanol-isopropanol; MS1, mass spectrometry survey scan; MS2/MS3, tandem mass spectrometry fragmentation experiments.

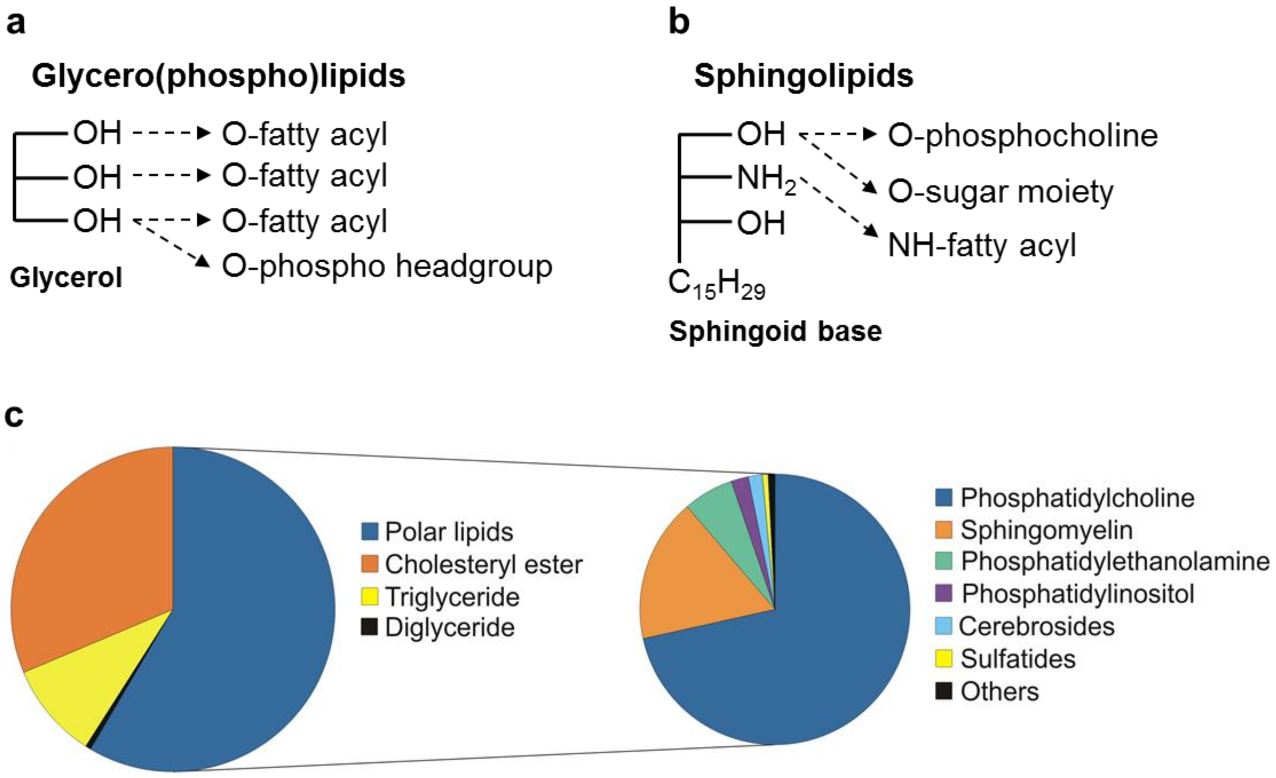

Supplementary Fig. S2. Basic lipid strutures and lipid class composition of CSF. Backbone structures and substitution possibilities of (**a**) glycero- and glycerophospholipids and (**b**) sphingolipids. (**a**) In glycerolipids, one to three hydroxyls of the glycerol are esterified with hydrophobic fatty acyl tails, whereas in glycerophospholipids, in addition to fatty acyls, one of the hydroxyls is linked to a different phosphate-containing headgroup, e.g., phosphocholine in phosphatidylcholine. (**b**) Sphingolipids contain a sphingoid base backbone. In ceramide, the amino group of the backbone is esterified with a fatty acyl, whereas sphingolipids upstream of ceramide might additionally contain phosphocholine as in sphingomyelin or different sugar moieties as found in cerebrosides, sulfatides and gangliosides. (**c**) Composition (mol%) of the whole CSF lipidome (left pie) and that of the CSF polar lipidome (right pie) based on quantified lipid classes. We note that higher gangliosides (GM1, GD, GT) and cholesterol, which represent remarkable contributions to the CSF lipidome (ca. 10% each, see Ref 17 in the main text), were not assessed in the present study.

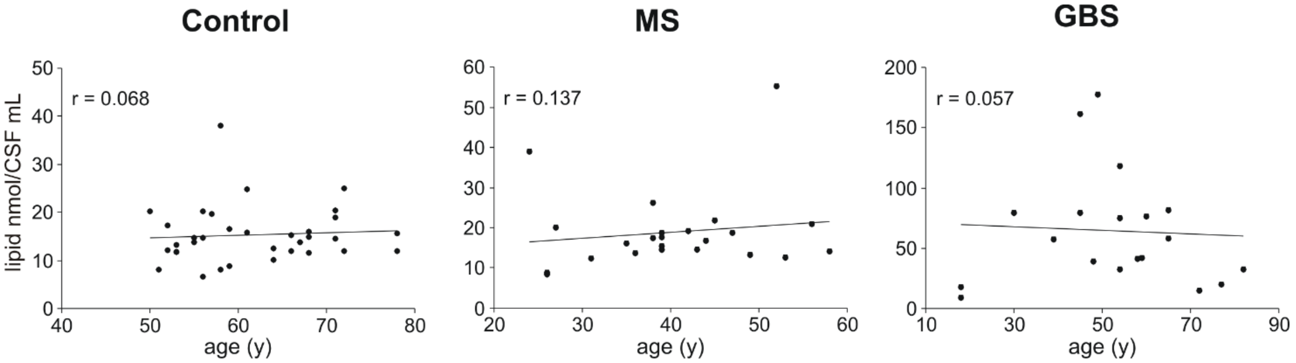

Supplementary Fig. S3. Correlation between patient age and total lipid content. Circles in scatter plots represent individual samples; control, n = 34; MS, n = 24; GBS, n = 19. Solid lines represent linear regression. r values represent Pearson correlation coefficients. Control, non-demyelinating diseases; MS, multiple sclerosis; GBS, Guillain–Barré syndrome.

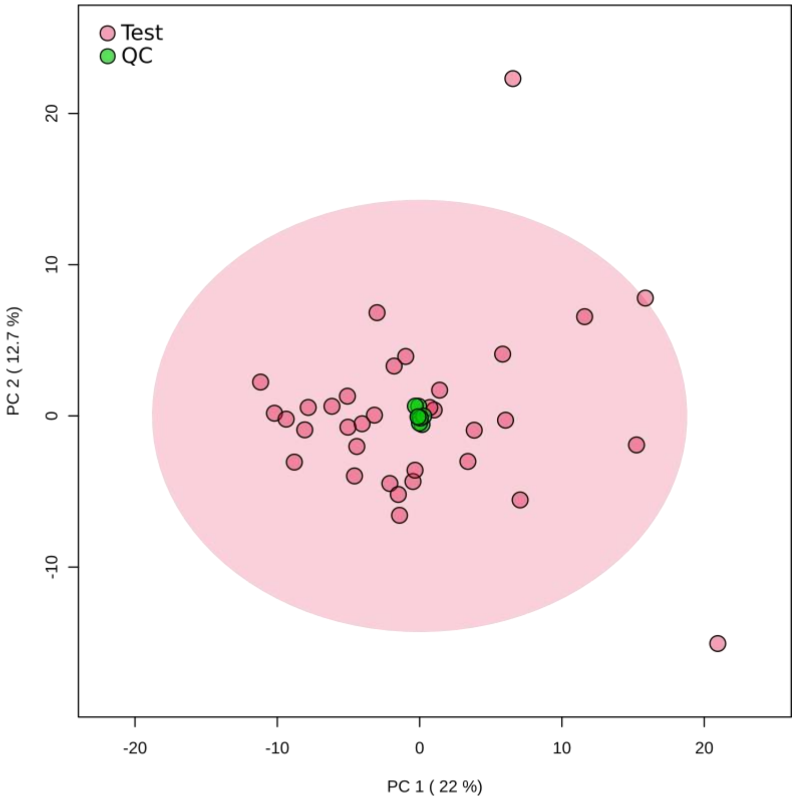

Supplementary Fig. S4. PCA scores plot of test and injection QC samples. Individual control samples were considered as test samples, whereas the QC sample was prepared by pooling the control samples. PCA, principal component analysis.
